# Resection of large terminal ileum polyp: usefulness of underwater EMR

**DOI:** 10.1016/j.vgie.2023.07.010

**Published:** 2023-10-05

**Authors:** Satoki Shichijo, Yasuhiro Tani, James Weiquan Li, Yoji Takeuchi, Noriya Uedo

**Affiliations:** 1 Department of Gastrointestinal Oncology, Osaka International Cancer Institute, Osaka, Japan; 2 Department of Gastrointestinal Oncology, Osaka International Cancer Institute, Osaka, Japan; 3 Department of Gastroenterology and Hepatology, Changi General Hospital, Singapore Health Services, Singapore; 4 Department of Gastrointestinal Oncology, Osaka International Cancer Institute, Osaka, Japan; 5 Department of Gastroenterology and Hepatology, Gunma University Graduate School of Medicine, Maebashi, Gunma, Japan; 6 Department of Gastrointestinal Oncology, Osaka International Cancer Institute, Osaka, Japan

## Abstract

Video 1 Underwater endoscopic mucosal resection for a large polyp at the terminal ileum.

Underwater endoscopic mucosal resection for a large polyp at the terminal ileum.

Underwater EMR (UEMR), first described by Binmoeller et al^1^ in 2012, has been studied extensively for colorectal^2^^,^^3^ and duodenal tumours.^4^^,^^5^ However, the use of UEMR for small intestinal polyps remains limited, and its usefulness remains unknown.

A 70-year-old man underwent follow-up positron emission tomography after a wide tumor resection of a malignant soft tissue gluteal tumor. He had no other history. An enhancing lesion was observed in the terminal ileum. A colonoscopy confirmed a 20-mm Paris Ip polyp (Fig. 1). Magnified narrow-band imaging showed a tumor pattern on top of the polyp (Fig. 2), while a villous pattern was noted at its stalk. However, the narrow lumen of the terminal ileum and scope position made optimal positioning for visual inspection of the entire polyp technically challenging. A biopsy from the top revealed an intestinal-type adenoma without signs of invasion, and UEMR was performed (Video 1, available online at www.videogie.org).

**Figure 1:**
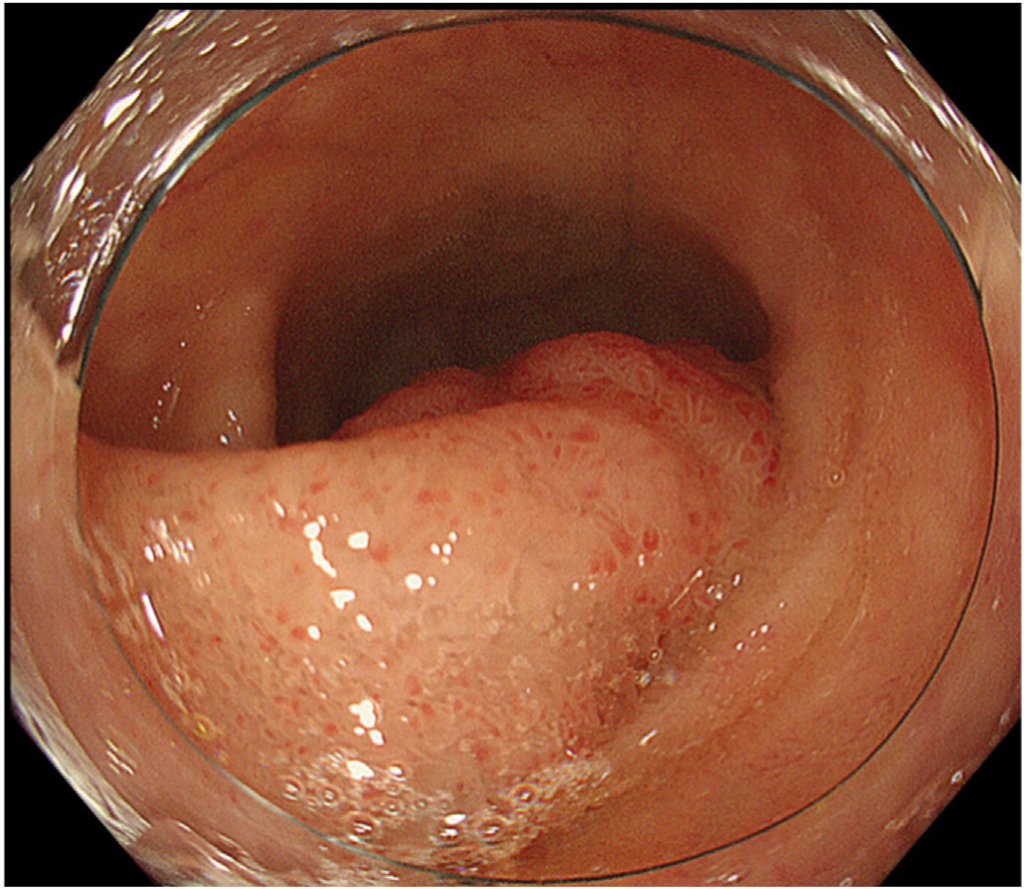
A 20-mm Ip polyp at the terminal ileum.

**Figure 2:**
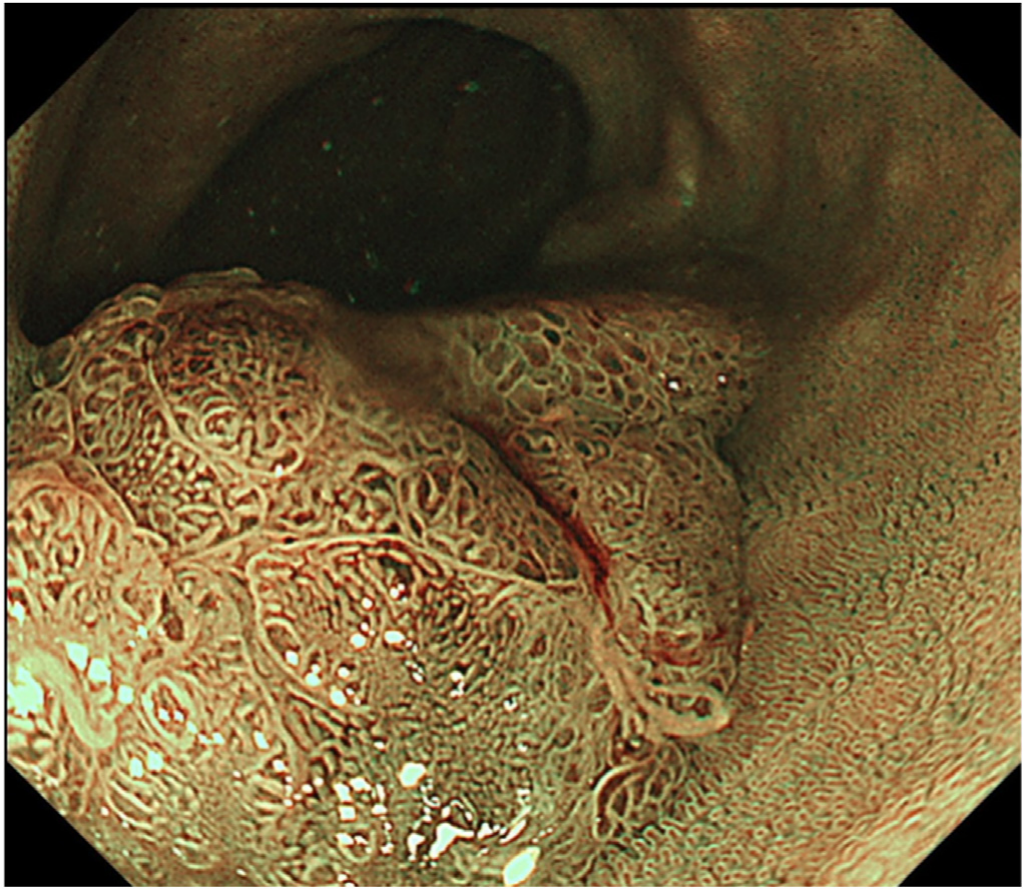
Magnified narrow-band imaging showed a tumor pattern at the top of the polyp.

The lumen of the terminal ileum was infused with normal saline through the waterjet of the colonoscope (Fig. 3). This made the polyp float and permitted thorough inspection despite the limited maneuverability of the colonoscope. Stalk ligation using an endoloop was facilitated with the underwater technique, hot snare polypectomy was performed, and an additional clip was placed to prevent delayed bleeding because once delayed bleeding occurs, control of the bleeding would be difficult in a narrow lumen in the terminal ileum. Pathological diagnosis was intramucosal adenocarcinoma in a tubulovillous adenoma, 0-Ip, tub1, pTis, ly0, v0, HM0, VM0 (Fig. 4).

**Figure 3:**
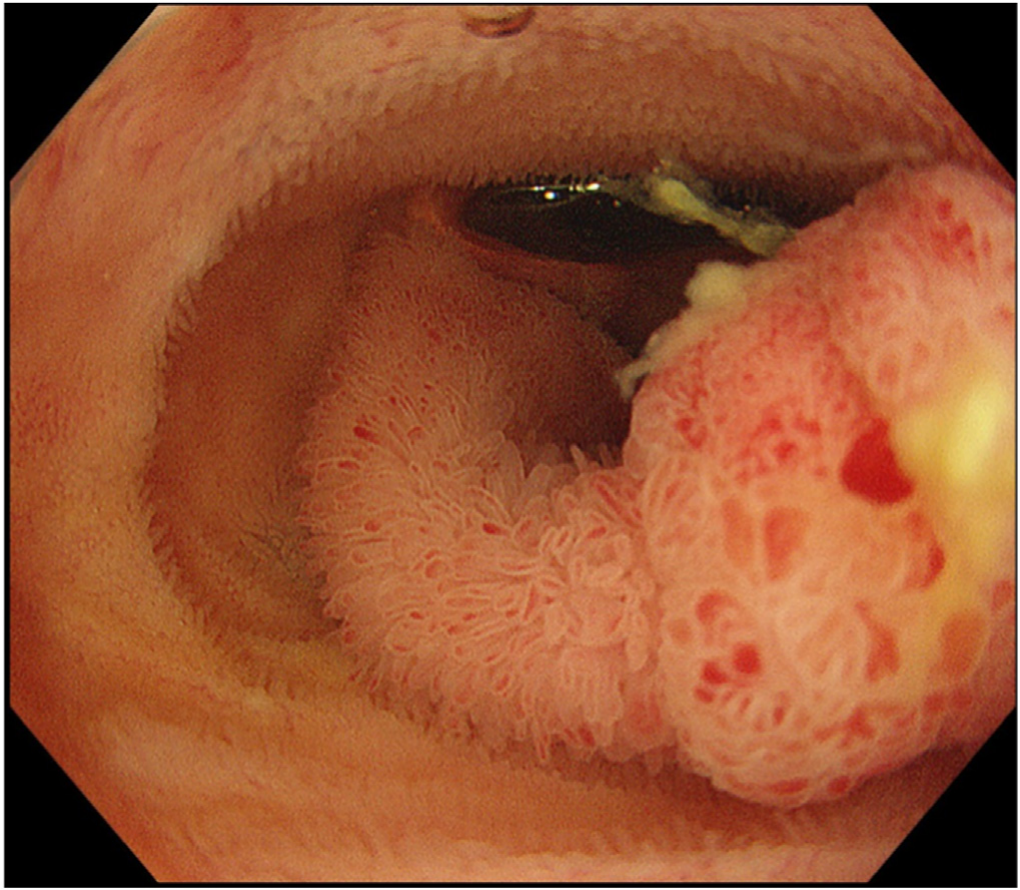
The polyp underwater.

**Figure 4:**
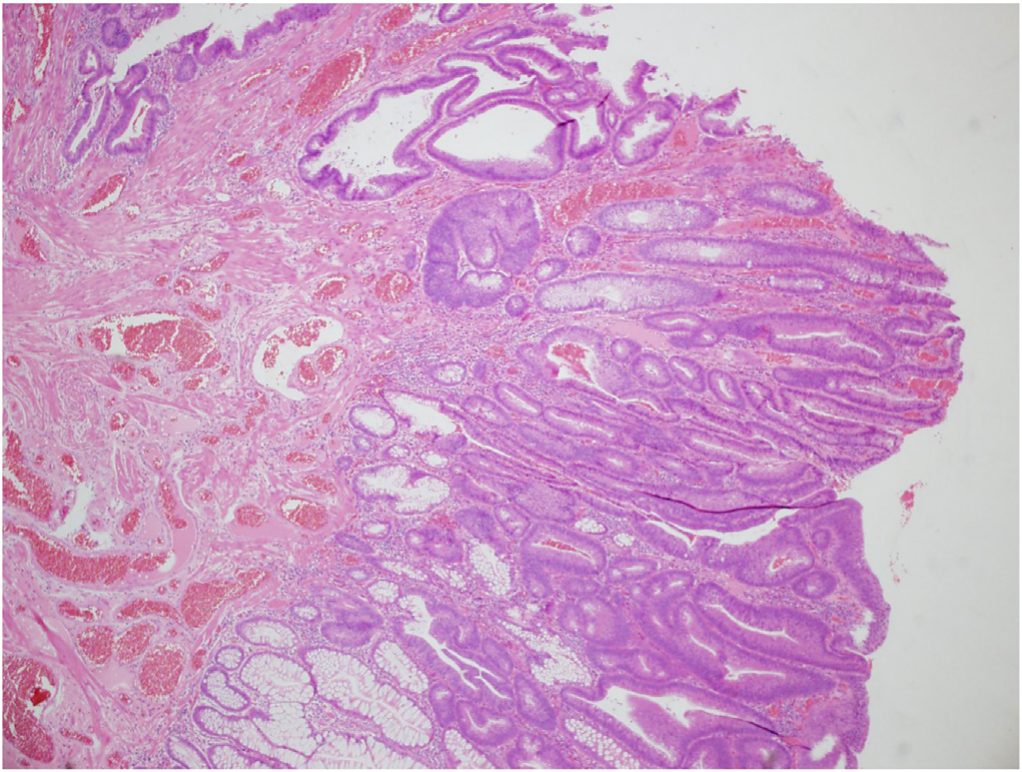
The pathologic diagnosis was an adenocarcinoma in a tubulovillous adenoma, 0-Ip, tub1, pTis, ly0, v0, HM0, VM0 (H&E, orig. mag. ×40).

Performing UEMR in the narrow lumen of the small intestine allows visualization and en bloc resection, resulting in a curative resection of adenocarcinoma in this patient.

## Disclosure

The authors did not disclose any financial relationships.

## Supplementary data

Video 1 Underwater endoscopic mucosal resection for a large polyp at the terminal ileum.
